# Correlation between alterations in cognitive function and mean severity of psychotic symptoms in patients diagnosed with schizophrenia spectrum disorders and its clinical application

**DOI:** 10.1192/j.eurpsy.2021.2143

**Published:** 2021-08-13

**Authors:** P. Malliaris, K. Bonotis

**Affiliations:** 1 Psychiatry, General Hospital of Karditsa, Karditsa, Greece; 2 Psychiatry, University of Thessaly, Larisa, Greece

**Keywords:** Schizophrenia spectrum disorders, cognitive assessment, Cognitive decline, Clinician-Rated Dimension of Psychosis Symptom Severity

## Abstract

**Introduction:**

Schizophrenia spectrum disorders (SSD) are characterized by heterogeneity. Cognitive decline, due to recent research results, appears to be a core symptom of schizophrenia. Dimensional approach of SSDs allows the separate assessment of each psychotic symptom, as well as cognitive functioning. Thus, correlations among them and their alterations, between baseline and follow up examination, can be estimated.

**Objectives:**

The objective of this study is to correlate observed alterations in cognitive performance in patients diagnosed with schizophrenia spectrum disorders, compared with baseline measurement, with alterations in severity of psychotic symptoms.

**Methods:**

85 Patients diagnosed with schizophrenia spectrum disorders, attended in the Outpatient Department of Early Intervention in Psychosis of University of Thessaly, Greece and its affiliated psychiatric clinics, were evaluated the last 24 months, using the CRDPSS (Clinician-Rated Dimensions of Psychosis Symptoms Severity) measure and the validated greek version of the MoCA test. 37 of them had a follow up evaluation. The relationship between the two new categorical variables [dMoCA (positive- negative) and dmCRDPSS7 (positive-negative)] was assessed with x² test.

**Results:**

Alterations in cognitive function, as assessed with MoCA scale and dMoCA variable, were inversely correlated with the alteration in mean severity of other dimensions of psychosis symptoms (dmCRDPSS7), x²(1, N = 37) = 9.4891, p = .0021.

**Conclusions:**

Our data suggest that alterations in cognitive performance may predict an inverse effect in the severity of psychotic symptoms. Periodic follow up of cognitive functioning in patients diagnosed with schizophrenia spectrum disorders is suggested, since it can be interpreted in clinically useful information considering relapse.

**Disclosure:**

No significant relationships.

